# Pain as bad as you can imagine or extremely severe pain? A randomized controlled trial comparing two pain scale anchors

**DOI:** 10.1186/s41687-023-00665-w

**Published:** 2023-11-29

**Authors:** Amy L. Tin, Mia Austria, Gabriel Ogbennaya, Susan Chimonas, Paulin Andréll, Thomas M. Atkinson, Andrew J. Vickers, Sigrid V. Carlsson

**Affiliations:** 1 Department of Epidemiology and Biostatistics, Memorial Sloan Kettering Cancer Center, New York, NY USA; 2 Department of Anaesthesiology and Intensive Care Medicine/Pain Centre, Region Västra Götaland, Sahlgrenska University Hospital/Östra, Gothenburg, Sweden; 3 Department of Anaesthesiology and Intensive Care Medicine, Institute of Clinical Sciences, Sahlgrenska Academy at University of Gothenburg, Gothenburg, Sweden; 4 Department of Psychiatry and Behavioral Sciences, Memorial Sloan Kettering Cancer Center, New York, NY USA; 5 Department of Surgery (Urology Service), Josie Robertson Surgery Center, Memorial Sloan Kettering Cancer Center, 1133 York Ave, New York, NY 10065 USA; 6 Department of Urology, Institute of Clinical Sciences, Sahlgrenska Academy at University of Gothenburg, Gothenburg, Sweden; 7 Department of Translational Medicine, Division of Urological Cancers, Medical Faculty, Lund University, Lund, Sweden

**Keywords:** Pain, Numeric rating scale, Validity, Patient-reported outcomes

## Abstract

**Background:**

A common method of pain assessment is the numerical rating scale, where patients are asked to rate their pain on a scale from 0 to 10, where 0 is “no pain” and 10 is “pain as bad as you can imagine”. We hypothesize such language is suboptimal as it involves a test of a cognitive skill, imagination, in the assessment of symptom severity.

**Methods:**

We used a large-scale online research registry, ResearchMatch, to conduct a randomized controlled trial to compare the distributions of pain scores of two different pain scale anchors. We recruited adults located in the United States who reported a chronic pain problem (> 3 months) and were currently in pain. Participants were randomized in a 1:1 ratio to receive pain assessment based on a modified Brief Pain Inventory (BPI), where the anchor for a score of 10 was either “extremely severe pain”, or the original BPI, with the anchor “pain as bad as you can imagine”. Participants in both groups also answered additional questions about pain, other symptomatology and creativity.

**Results:**

Data were obtained from 405 participants for the modified and 424 for the original BPI. Distribution of responses to pain questions were similar between groups (all *p*-values ≥ 0.12). We did not see evidence that the relationship between pain score and the anchor text differed based on self-perceived creativity (all interaction *p*-values ≥ 0.2). However, in the key analysis, correlations between current pain assessments and known correlates (fatigue, anxiety, depression, current pain compared to a typical day, pain compared to other people) were stronger for “extreme” vs. “imaginable” anchor text (*p* = 0.005).

**Conclusion:**

Pain rating scales should utilize the modified anchor text “extremely severe pain” instead of “pain as bad as you can imagine”. Further research should explore the effects of anchors for other symptoms.

**Supplementary Information:**

The online version contains supplementary material available at 10.1186/s41687-023-00665-w.

## Introduction

Two of the most common patient-reported outcomes (PRO) instruments to assess pain intensity are the numerical rating scale (NRS), where patients select a number from 0 to 10 corresponding to their experienced pain level, and the visual analog scale (VAS), where patients are asked to mark their pain level on a line (usually 100-mm) between two endpoints. Both the NRS and VAS include “anchor” text at each end. Whereas the anchor text for 0 is typically “no pain”, the anchor text for the highest pain level varies greatly [1] and often references imagination, whether explicitly (e.g. “worst pain imaginable”) or implicitly (e.g. “worst pain possible” or “pain as bad as it could be”). For example, in the commonly used Brief Pain Inventory [2] (BPI), used for pain assessment in both chronic and cancer-related pain populations [3, 4], the anchor for a pain score of 10 is “pain as bad as you can imagine”. This anchor text has been recommended to determine pain intensity of cancer patients at a consensus meeting on cancer pain assessment and classification in 2009 [4], though it is unclear what other anchor texts were considered or the rationale for this selection.

Pain as a symptom is a personal sensory experience [5]. Thus, clinicians depend on patient ability to self-report their experience, interpretation and imagination when describing their pain intensity. Therefore, the wording of pain scale anchors is critical when assessing a person’s pain. We hypothesize that anchors that involve explicit or implicit reference to imagination are suboptimal as they involve a test of a cognitive skill in the assessment of symptom severity. It seems reasonable to suppose that a patient with a vivid imagination might assign a lower pain rating than other patients experiencing similar levels of pain as they have access to a wider and more extreme range of “imaginable” levels of pain. Moreover, a scale that mixes a test of imagination with assessment of pain would be expected to have lower convergent validity, that is, it would correlate less strongly with other predictors of pain. Instead, we hypothesize that the alternative anchor “extremely severe pain”, commonly used in other pain instruments [6, 7], would have better psychometric properties. There is a dearth of studies comparing alternative language for a given psychometric instrument. We undertook a randomized controlled trial to compare an alternative anchor for the BPI pain intensity questions when administered to patients with chronic pain.

## Methods

The study was approved by the Institutional Review Board at Memorial Sloan Kettering Cancer Center.

### Recruitment

Participants were recruited from the online database ResearchMatch, a national health volunteer registry that was created by several academic institutions and supported by the United States National Institutes of Health (NIH) as part of the Clinical Translational Science Award (CTSA) program [8]. ResearchMatch has a large population of volunteers who have consented to be contacted by researchers about health studies for which they may be eligible. The total number of volunteers in the registry varies by day and was approximately 156,200 at the time the study was conducted. A total of 31,102 participants were randomly sampled from the ResearchMatch database and contacted for participating in the study, using a contact message e-mailed to participants in ResearchMatch. Individuals expressing interest in participating volunteered into the study through reviewing a study information sheet and were redirected to the online survey. The survey completion time took about 5 to 10 min.

Patients who fulfilled the following criteria were considered eligible for the study: any gender, location in the United States, age above 21, chronic pain problem (> 3 months) with current pain. Eligibility was confirmed using ResearchMatch demographics filters together with an eligibility screener verifying eligibility.

### Randomization

Randomization and automated survey data collection was embedded within the Research Electronic Data Capture (REDCap) software used to record responses, ensuring full allocation concealment [9, 10]. Subjects who expressed interest in participating in the study were randomized (1:1) to one of two groups with differing anchor text for the four BPI items on the pain intensity domain: (A) Questionnaire A (intervention): scale from 0 (“no pain”) to 10 (modified anchor: “extremely severe pain”) or (B) Questionnaire B (control): scale from 0 (“no pain”) to 10 (original anchor: “pain as bad as you can imagine”). In addition to the four questions from the BPI (pain at its worst, least, on average, right now), we also asked patients to rate pain on a typical day during a time when pain problem was troubling. Patients were also asked for their age, pain in the last 24 h compared to a typical day (1 question), pain compared to other people with the same pain problem (1 question), pain location (from Breivik questionnaire [11]), fatigue, anxiety, depression (3 questions, Edmonton Symptom Assessment System [12]), and creativity (2 questions, Kumar and Holman’s Global Measure of Creativity Capacity [13]). Written permission to use and adapt these questionnaires for research was sought from the copyright holders. The full text of questions asked to patients are shown in Supplementary Tables 1, as well as the short-hand reference term used throughout this paper.

As the majority of questions are phrased such that higher values correspond to worse symptoms, the two questions related to pain “compared to typical day” and “compared to other people” were reverse coded to match the other questions. In particular, responses for both questions originally ranged from 1 corresponding to “much worse” to 5 corresponding to “much better” and were rescaled to be subtracted from 6, so that the updated values would be on the scale of 1 corresponding to “much better” to 5 corresponding to “much worse.”

Imagination is key for creative thought [14]. We hypothesize that participants with vivid imagination will assign a lower pain rating than others as they can imagine a wider and more extreme range of “imaginable” levels of pain. The two creativity questions in the Kumar and Holman’s Global Measure of Creativity Capacity were chosen based on a literature review of validated surveys and was determined suitable for our study because of low survey burden (two items) and high reliability (alpha 0.76) for perceived creativity [13]. The first question asks whether participants considered themselves to be a creative person, and the other question asks whether participants are engaged in creative type work on a regular basis, have answer options ranging from 1 “strongly agree” to 5 “strongly disagree.” Each participant was assigned a creativity score calculated as the sum of the two responses subtracted from 10. As such, the creativity score ranged from 0 to 8 with higher values corresponding to greater creativity.

### Statistics

We were first interested in assessing whether there was a difference in the distribution of responses to individual questions with differing anchor text based on randomization group. The distribution of answers to each of the pain questions, by group, were visualized using raincloud plots. We further presented a table reporting the mean (standard deviation) response to the pain questions based on randomization group, the difference in means between the two groups, and the 95% confidence interval around the difference, as well as the *p*-value. To ensure we did not miss any distributional differences, we additionally dichotomized (worst ≥ 9, least ≤ 7, average ≥ 8, right now ≥ 8, troubling = 10) and categorized (0–1 vs. 2–4 vs. 5 vs. 6–8 vs. 9–10) responses options and evaluated whether there were differences in proportions between the two groups. Finally, we presented the median and quartiles and tested for differences in the distribution of responses to the questions between groups. The aforementioned analyses were limited to the five pain questions which had differing anchor text between the two questionnaires, as the remaining questions were asked unrelated to the anchoring text. Comparison of means was using the Welch Two Sample t-test; comparison of dichotomized responses was using Pearson’s Chi-squared test, categorized responses were using Pearson’s Chi-squared test and Fisher’s exact test, and continuous responses using Wilcoxon rank sum test.

Our key analysis concerned the correlation by randomization group between the 5 pain questions related to the anchoring text, the other two pain questions unrelated to the anchoring text (“compared to a typical day” and “compared to other people”) and the correlates of pain: fatigue, depression and anxiety. We tested whether there was an overall difference in correlations between the two groups using a permutation test, and if there was evidence of a difference, we then tested the difference in individual question pairs based on group by using Fisher’s r-to-Z transformation.

Finally, we were interested in assessing whether the association between pain score and anchor text differed based on creativity. For each of the five pain questions, we created separate linear regression models with the individual pain score as the outcome, the randomization group and creativity score as the primary predictors and an interaction term between the two predictors.

We chose a sample size of 1,000 for practical reasons of feasibility and cost. We prespecified feasibility as being able to obtain an 80% response rate within a reasonable timeframe (target < 3 months). We prespecified that our cohort would constitute a group of chronic pain patients with elevated pain scores if the mean score for “worst pain” was greater than 6 (with similar standard deviation between the two groups). All analyses were conducted using R version 4.1.0 with the raincloudplots (v0.2.0), tidyverse (v1.3.1) and gtsummary (v1.5.0) packages.

## Results

Of 31,102 participants who received a contact message between June and October 2021, 1,133 expressed interest in participating in the study. Of those, 1,107 initiated the eligibility screener, 2 did not complete the screener and 99 were considered ineligible (n = 76 reported not suffering from pain from an illness or medical condition, n = 17 reported suffering from pain due to illness of medical condition ≤ 3 months, n = 5 not located in the U.S., n = 1 did not answer whether they experienced pain > 3 months). Of the remaining 1,006 participants randomized to the two version of the surveys, 829 completed the surveys, yielding a completion rate of 82%, thus reaching over the prespecified 80% response rate. Completion rates were similar between the two groups: 405/501 (81%) among those randomized to the (modified) version of the BPI where the anchor was updated to “extremely severe pain” (Questionnaire A) and 424/505 (84%) among those randomized to the (original) version of the BPI with the anchor of “pain as bad as you can imagine” (Questionnaire B).

Table 1 presents the participant characteristics and responses to the questions unrelated to the anchoring text. The median age in our patient group was 53 (IQR 38, 64) and predominately (78%) female. There was an unexpectedly high reporting of creativity, with three-quarters of participants considering themselves creative persons, and just under half (48%) reporting creative work on a regular basis. Both groups reported average pain intensity NRS 5 and worst pain intensity NRS 7 (in the last 24 h), with no differences between the groups (Supplementary Table 3c). Supplementary Table 2 presents the proportion of participants with pain in specified body parts, where the median number of pain locations was similar between groups: 9 (IQR 5, 14) in the extreme pain group and 9 (IQR 5, 14) in the imaginable group. Median scores on a 0–10 scale were similar between anchor groups for fatigue (6 [IQR 4, 8]), anxiety (3 [IQR 2, 6]) and depression (3 [IQR 1, 6]) (Table 1).

**Table 1:** Characteristics and responses to questions. Data presented as frequency (percentage) and median (quartiles)

	N	Extreme, N = 405	Imagine, N = 424
Age	829	53 (37, 63)	54 (38, 65)
Gender	829		
Male		81 (20%)	87 (21%)
Female		316 (78%)	327 (77%)
Another term		8 (2.0%)	10 (2.4%)
Pain compared to typical day	828		
Much Better		29 (7.2%)	27 (6.4%)
Somewhat Better		85 (21%)	85 (20%)
About the Same		181 (45%)	206 (49%)
Somewhat Worse		92 (23%)	98 (23%)
Much Worse		17 (4.2%)	8 (1.9%)
Pain compared to other people	820		
Much Better		25 (6.2%)	20 (4.8%)
Somewhat Better		88 (22%)	91 (22%)
About the Same		175 (44%)	209 (50%)
Somewhat Worse		91 (23%)	85 (20%)
Much Worse		22 (5.5%)	14 (3.3%)
Fatigue (0–10)	828	6 (4, 8)	6 (4, 8)
Anxiety (0–10)	827	3 (2, 6)	3 (1, 6)
Depression (0–10)	829	3 (1, 6)	3 (1, 6)
Creative person	825		
Strongly Agree		133 (33%)	112 (27%)
Agree		177 (44%)	194 (46%)
Unsure		40 (9.9%)	46 (11%)
Disagree		40 (9.9%)	52 (12%)
Strongly Disagree		13 (3.2%)	18 (4.3%)
Creative work	828		
Strongly Agree		58 (14%)	51 (12%)
Agree		146 (36%)	141 (33%)
Unsure		53 (13%)	37 (8.7%)
Disagree		107 (26%)	141 (33%)
Strongly Disagree		41 (10%)	53 (13%)
Creativity Score	825	6 (4, 7)	5 (3, 6)

**Fig. 1:**
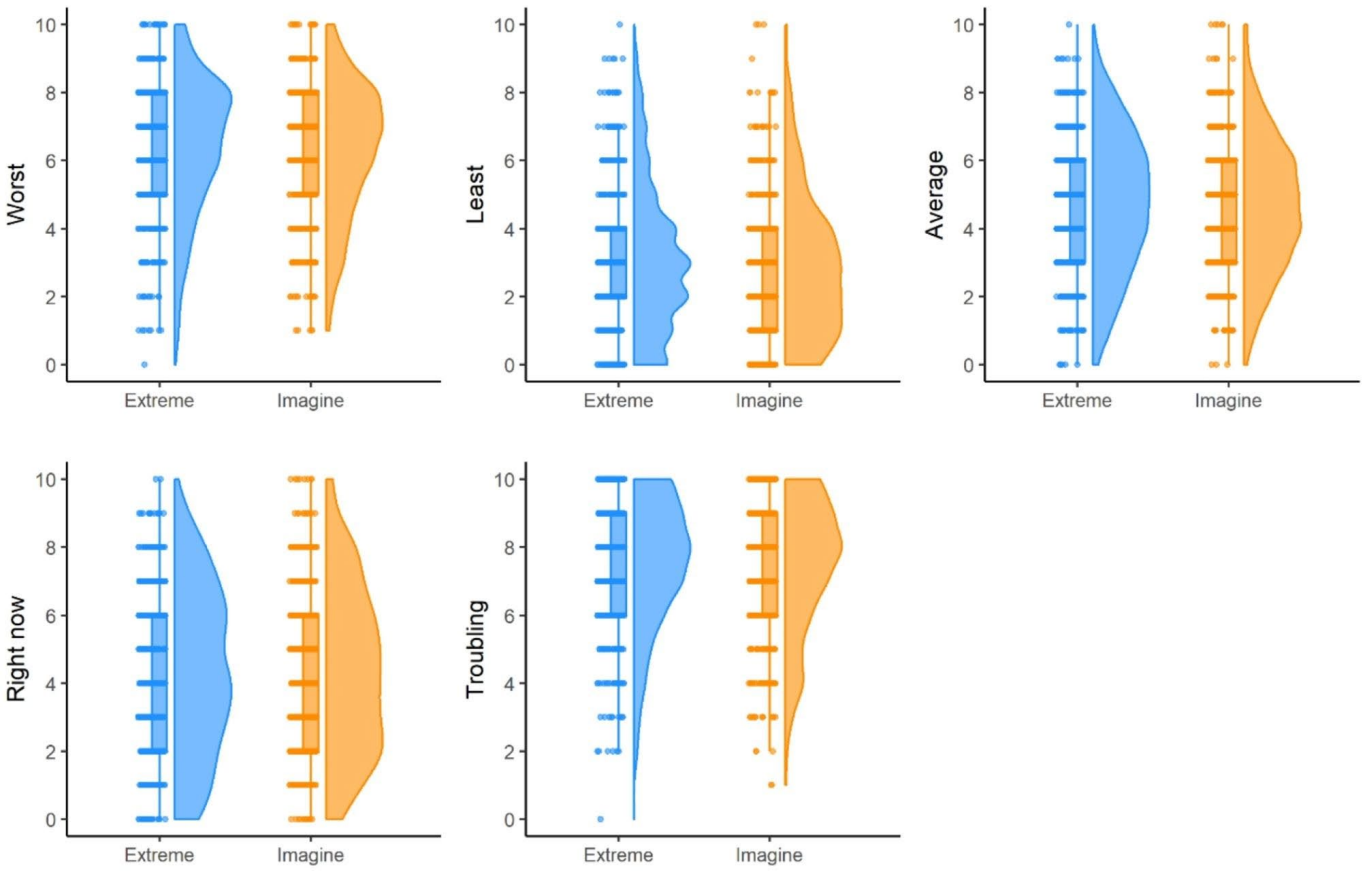
Rain-cloud plots for pain questions based on anchor group (“*Extreme*” corresponds to the changed anchor text “*Extremely severe pain*”; “*Imagine*” corresponds to the original anchor text “*Pain as bad as you can imagine*”)

The similarity in participant responses is depicted in Fig. 1. For each of the five pain questions, responses to each of the questions were fairly consistent, regardless of randomization group. Table 2 presents the mean (standard deviation) of responses to the five pain questions by randomization group. Of particular interest, the mean worst pain intensity was over NRS 6, hence our population is one with clinically significant pain. We did not see evidence of differences in the mean response to any of the five pain questions (all *p*-values ≥ 0.12), with the largest difference of under a quarter of a point (corresponding to the question “least” pain), a very small effect for an 11-point scale. Evaluation of responses to the five questions utilizing different measures are shown in Supplementary Tables 3a-3c, where we likewise did not see evidence of a difference in responses to questions based on randomization group.

**Table 2:** Responses to pain questions based on anchor group (“*Extreme*” corresponds to the changed anchor text “*Extremely severe pain*”; “*Imagine*” corresponds to the original anchor text “*Pain as bad as you can imagine*”). Data presented as mean (standard deviation)

	Extreme, N = 405	Imagine, N = 424	Difference	95% CI	*p*-value
Worst	6.45 (2.01)	6.30 (1.96)^1^	0.15	-0.12, 0.42	0.3
Least	3.07 (2.18)	2.85 (2.08)^1^	0.23	-0.06, 0.52	0.12
Average	4.75 (2.05)^1^	4.69 (1.94)^1^	0.06	-0.22, 0.33	0.7
Right now	4.34 (2.48)	4.36 (2.44)^2^	-0.02	-0.36, 0.32	> 0.9
Troubling	7.50 (1.92)	7.42 (1.98)	0.07	-0.19, 0.34	0.6

^1^1 unknown response. ^2^2 unknown responses

**Fig. 2:**
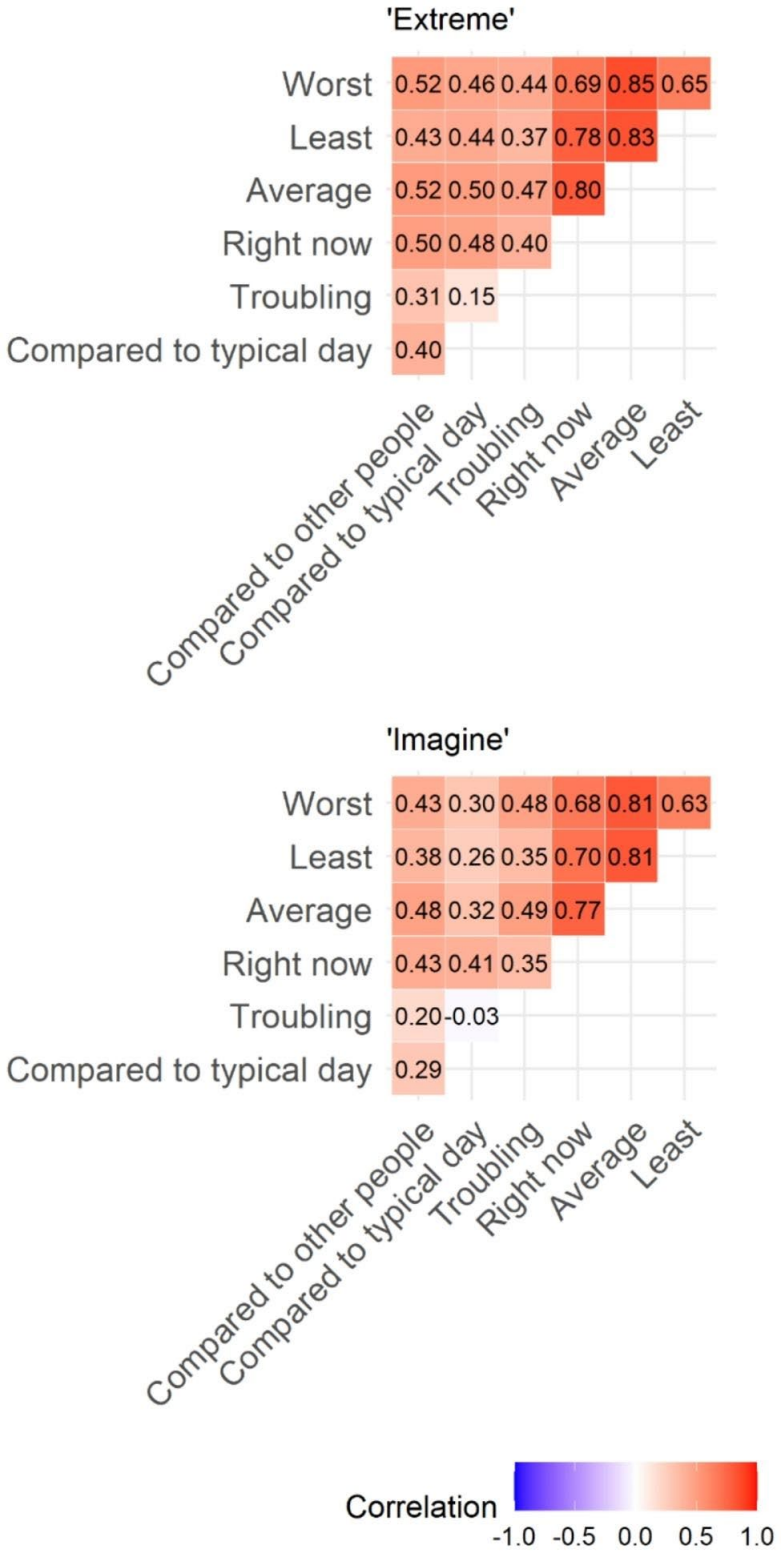
Correlation between pain questions based on anchor group (“*Extreme*” corresponds to the changed anchor text “*Extremely severe pain*”; “*Imagine*” corresponds to the original anchor text “*Pain as bad as you can imagine*”)

Figure 2 presents the correlations between each pair of the pain questions based on randomization group. The strength of the correlations were consistent regardless of randomization group, for example the highest correlations were between the question pairs “worst” and “average,” and “least” and “average,” and the lowest correlations existed between the question pair “troubling” and “compared to a typical day”.

Omitting the pain question “troubling” which is a recall question, Cronbach’s alpha between the four pain questions was 0.92 among surveys with the anchor text “pain as bad as you can imagine” and 0.93 among surveys with the anchor text changed to “extremely severe pain.” Overall, correlations between the pain questions and known correlates were stronger for “extreme” vs. “imaginable” anchor text (*p* = 0.005). Analyses evaluating whether the correlation between the pain questions and pain “compared to other people” or “compared to a typical day” differed based on the anchoring text are shown in Table 3. We saw non-significant stronger correlations between the pain questions and pain “compared to other people” in the “extremely severe pain” anchor group compared to the “pain as bad as you can imagine” group (overall *p*-value = 0.3) (Table 3). Excluding the question “right now”, we saw evidence of a difference in correlations between the main pain questions and pain “compared to a typical day” (all *p*-values ≤ 0.007), again with stronger correlations between questions among the group with the anchor text avoiding reference to imagination (Table 3).

**Table 3:** Correlation between pain questions and “Compared to other people” and “Compared to typical day” and whether correlations differ based on anchor group (“*Extreme*” corresponds to the changed anchor text “*Extremely severe pain*”; “*Imagine*” corresponds to the original anchor text “*Pain as bad as you can imagine*”)

	Compared to other people		Compared to typical day	
	Extreme	Imagine	Extreme	Imagine
Worst	0.52	0.43	0.46	0.30
Least	0.43	0.38	0.44	0.26
Average	0.52	0.48	0.50	0.33
Right now	0.50	0.43	0.48	0.41

Table 4 presents the correlation between the pain questions and the fatigue, anxiety and depression questions based on randomization group, where there was significantly stronger correlation between pain questions and depression among patients with the anchor text “extremely severe pain” (overall *p*-value = 0.038; testing for a difference in the correlation between worst pain and depression based on anchor did not meet conventional levels of significance). We saw non-significantly stronger correlation between pain questions and fatigue (overall *p*-value = 0.12) and anxiety (overall *p*-value = 0.2) among patients with the anchor text “extremely severe pain”. Supplementary Table 4 presents the correlation between the pain questions and age and sex based on randomization group. As expected, correlations were very weak and did not differ between groups.

**Table 4:** Correlation between pain questions and fatigue, depression and anxiety questions based on anchor group (“*Extreme*” corresponds to the changed anchor text “*Extremely severe pain*”; “*Imagine*” corresponds to the original anchor text “*Pain as bad as you can imagine*”)

	Fatigue		Depression		Anxiety	
	Extreme	Imagine	Extreme	Imagine	Extreme	Imagine
Worst	0.39	0.33	0.36	0.27	0.35	0.27
Least	0.41	0.29	0.45	0.28	0.42	0.30
Average	0.47	0.37	0.44	0.32	0.41	0.34
Right now	0.49	0.39	0.40	0.29	0.40	0.34

Supplementary Table 5 presents the estimates for the interaction terms between anchor group and creativity, where we did not see evidence of a difference in the association between creativity and pain based on anchor group (all interaction *p*-values ≥ 0.2). Analyses without the interaction term, showed no evidence of an association between creativity score and pain score or between anchor group and pain score (all *p*-values ≥ 0.15).

## Discussion

We compared two different anchors for the extreme high pain score for an NRS. Scores using the anchor “extremely severe pain” had stronger associations with known correlates of pain than when using the anchor “worst pain imaginable” without changing average pain scores. This relationship was unaffected by self-reported creativity. Although there has been considerable research on different pain scales - VAS, verbal rating scales, and NRS – there has been little work related to the differing anchor texts. In reviewing currently available pain outcomes measures, the Initiative on Methods, Measurement, and Pain Assessment in Clinical Trials (IMMPACT) found that different methods to all be reliable and valid, but no scale was consistently better [3]. The pain intensity NRS was found to be less abstract and easier to understand than other scales and studies using this scale tended to have less missing and incomplete data [3]. To enable consistency among studies, the IMMPACT initiative therefore recommends using an 11-point NRS (pain from 0 to 10) with the anchor text for the upper limit being “pain as bad as you can imagine” (from Cleeland and Ryan), but without any further rationale [3]. In a review of 43 articles examining various response scale selections, Safikhani et al. found that the pain NRS was the most frequently recommended due to it being simple, straightforward, easy to administer and interpret, being preferred by patients, leading to more complete responses, and having better psychometric properties [15]. They note that in addition to selection of the response scale, there are other complicating factors which should be considered, such as the exact wording of the response anchor, though they did not go on to examine the anchors, instead only focusing on the various response scales. In a separate review of 54 studies, Hjermstad et al. compared NRS, verbal rating scales, and/or VAS. Although they found that 24 different descriptors were used for the anchor labels, no comparisons of the texts for the extreme values were carried out, though they did speculate that the anchor text would influence responses, and thus called for additional research [1].

We did find one study, by Seymour et al., which explicitly compared different anchor texts (referred to as “end-phrase” throughout their paper) on VAS. Limited to 100 patients with dental pain, Seymour et al. compared the following four anchor texts: “troublesome”, “miserable”, “intense”, and “unbearable” to “worst pain imaginable” and saw that as the anchor became more extreme, the more the histogram would shift towards lower scores. Considering the shape of the histogram, combined with prompting the fewest observations at the extremes, they concluded that the text “worst pain imaginable” was the most suitable anchor. There were 14 responses at the extreme for the anchor of “worst pain imaginable” compared to the next lowest value of 18 (anchor text “troublesome”) for the question asking about present pain, and 6 compared to the frequency of 11 (anchor text “intense” and “unbearable”) when asking about worst pain [16]. However, the authors did not report whether these findings were statistically significant. Additionally, it is unclear why the ceiling effects should be the primary determinant of questionnaire wording and is questionable whether a ceiling effect was demonstrated. It is likely that there are patients who would never rate their pain as equal to “worst pain imaginable” regardless of how severe their dental pain was and would therefore respond below the maximum score on a given pain scale. In our cohort, we saw a non-significantly lower percentage of patients in that imagine group who reported pain at the extremes to the question asking about worst pain (11% vs. 13%, *p*-value = 0.4) and pain right now (16% vs. 20%, *p*-value = 0.2) and did not see any evidence of difference in the distribution of responses based on anchor text (Supplementary Tables 3a-3c).

Another common area of focus regards potential cutoffs to define severe pain. Boonstra et al. reported the cutoffs of ≤ 5 for mild, 6–7 for moderate, and ≥ 8 for severe pain in patients with chronic musculoskeletal pain [17] and Serlin et al. report similar cutoffs: 1–4 for mild: 5–6 for moderate, and 7–10 for severe pain in patients with cancer. We anticipate that one argument against the use of alternative anchor text is that it may require modification of any established cutoffs, be they used in clinical practice, or comparison of scores collected historically. While our current study does not delve into cut-points, one of our key results is that there were no differences in the distribution of responses to the individual questions asking about pain based on the two anchoring texts. We therefore have no reason to believe that use of the alternative anchor text “extremely severe pain” instead of the anchor text “pain as bad as you can imagine” or “worst pain possible” would modify established cut-points.

The participants in our study coincide with what would be expected for chronic pain patients. Breivik and colleagues conducted a cross-sectional telephone survey of nearly 9,000 people in 15 European countries and Israel and estimates the prevalence of chronic pain (≥ 6 months) in adults to be approximately 19%. Among those with chronic pain, respondents reported their pain intensity when they last experienced pain ≥ 5. In further interviews with nearly 5,000 people with chronic pain, the average age was 50 and 56% were females [11]. In our study, patients had a mean score of 5 in response to the question asking about average pain and the mean age in our cohort was similar (51 years old), however, our cohort had a larger proportion of female participants (78%). This can be explained in part by the fact that there are twice as many female volunteers in ResearchMatch than males [8], but nonetheless, our distribution of female participant does coincide with the 72% of females in Boonstra’s study of patients with chronic musculoskeletal pain [17] as well as data from the Swedish Quality Register for Pain Rehabilitation which comprises of 74% females [18].

This study is not without limitations. First, we accrued participants by appealing to volunteers in an online research registry with access to email who were redirected to an online survey. Therefore, it is unclear what types of selection bias may apply to our cohort. However, this methodology likely led to our sample being more representative of the general United States pain population, compared to participants had they been accrued from the clinics of the sort of tertiary care centers where psychometric research is often conducted. Additionally, due to the randomized design of our study, any bias from the accrual method would be balanced between the two groups answering the questionnaire based on differing anchoring text. A second limitation to our study is that participants were randomized to separate surveys with one of the two anchor texts (between-subject randomization) rather than to the order of which survey with the anchor text they would first complete followed by the survey with the second anchor text (within-subject randomization). This would have doubled the number of surveys available for analysis and responses to both questionnaires would have been correlated, allowing us to test for minor differences in responses to the individual questions. However, this would have been at the cost of potential contamination (responses to the second survey being influenced by what subjects saw in the first survey). Another limitation of our study is that it is possible that, had we used another measure of creativity, our hypothesis that there would be differences in the relationship between anchor text and pain level based on self-perceived creativity may have been supported. Among our cohort, we found that 75% of participants considered themselves as being a creative person and nearly half of participants agreed that they were engaged in creative work on a regular basis. However, both of these estimates are high, suggesting that our measures of creativity were not particularly discriminatory. Nonetheless, rejection of the null (which we were unable to do in this study), would only further support the use of the “extremely severe pain” anchor text. Lastly, participants in our study were only reading and completing the questionnaire in English. While we would not anticipate issues around translations of the anchors in different languages, as with the BPI and other pain NRS being validated in various languages, this could be an area for further research. We also recommend additional studies examining a wider variety of anchor texts which may include cognitive interviewing as areas of future study.

Our study is a rare example of comparative psychometric research. Normally, psychometric research is done on an individual instrument, with intra-item correlations, and correlations between scores and other patients’ aspects, calculated for a single instrument to determine whether it is psychometrically valid. Seldom do researchers compare the wording used in similar PRO instruments to determine which is better. In our current study, both approaches to pain assessment would be described as “valid” separately, but we found that the one with the anchoring text “extremely severe pain” has better properties than the other with the anchoring text “pain as bad as you can imagine.” This finding is only possible based on the randomized design. It is of note that studies comparing slight variations in language for scales need large samples. For instance, if the correlation between scale A and a variable is 0.45 but 0.3 for scale B, then a sample size of about 1,000 patients is needed for 80% power. Therefore, such studies have only recently been made feasible due the availability of electronic PROs and internet panels.

## Conclusion

Pain rating scales should use the anchor text “extremely severe pain” or anchor text that does not refer, explicitly or implicitly, to imagination, for instance, “worst pain imaginable”. Further research should explore the effects of anchors on NRS for other symptoms.

### Electronic Supplementary Material

Below is the link to the electronic supplementary material.

**Supplementary Material 1: Supplementary Table 1.** Full question text on questionnaire and shorthand reference used in text

**Supplementary Material 2: Supplementary Table 2.** Body Part where participants have pain. Data presented as frequency (percentage)

**Supplementary Material 3: Supplementary Table 3a**. Dichotomized responses to pain questions based on anchor group. (“*Extreme*” corresponds to the changed anchor text “*Extremely severe pain*”; “*Imagine*” corresponds to the original anchor text “*Pain as bad as you can imagine*”). Data presented as frequency (percentage). **Supplementary Table 3b**. Categorized responses to pain questions based on anchor group. (“*Extreme*” corresponds to the changed anchor text “*Extremely severe pain*”; “*Imagine*” corresponds to the original anchor text “*Pain as bad as you can imagine*”). Data presented as frequency (percentage). **Supplementary Table 3c**. Responses to pain questions based on anchor group (“*Extreme*” corresponds to the changed anchor text “*Extremely severe pain*”; “*Imagine*” corresponds to the original anchor text “*Pain as bad as you can imagine*”). Data presented median (quartiles)

**Supplementary Material 4: Supplementary Table 4.** Correlation between pain questions and age and sex questions based on anchor group (“*Extreme*” corresponds to the changed anchor text “*Extremely severe pain*”; “*Imagine*” corresponds to the original anchor text “*Pain as bad as you can imagine*”)

**Supplementary Material 5: Supplementary Table 5.** Interaction term from linear regression model with pain as the outcome, primary predictors of creativity score, anchor group (“*Extreme*” corresponds to the changed anchor text “*Extremely severe pain*”; “*Imagine*” corresponds to the original anchor text “*Pain as bad as you can imagine*”), and interaction between anchor group and creativity score

## Data Availability

The datasets used and/or analyzed during the current study are available from the corresponding author on reasonable request.
