# Supplementary material for: Pain as bad as you can imagine or extremely severe pain? A randomized controlled trial comparing two pain scale anchors
Source: J Patient Rep Outcomes. 2023 Nov 29;7:123. doi: 10.1186/s41687-023-00665-w (PMC10686922; doi:10.1186/s41687-023-00665-w)
Supplement: Supplementary file 1 — Supplementary Material 1: Supplementary Table 1. Full question text on questionnaire and shorthand reference used in text [file 41687_2023_665_MOESM1_ESM.docx]

**Supplementary Table 1.** Full question text on questionnaire and shorthand reference used in text.

| **Question Text** | **Shorthand reference** |
| --- | --- |
| Please rate your pain by selecting the number that best describes your pain at its **worst** in the last 24 hours | Worst |
| Please rate your pain by selecting the number that best describes your pain at its **least** in the last 24 hours | Least |
| Please rate your pain by selecting the number that best describes your pain on average in the last 24 hours | Average |
| Please rate your pain by selecting the number that tells how much pain you have right now | Right now |
| Think about a time in your life when your pain problem was troubling you. How would you rate your pain during a typical day during that time? | Troubling |
| Compared to my typical day, my pain in the last 24 hours has been | Compared to a typical day |
| Compared to other people who have the same pain problem as I do, I consider my pain to be | Compared to other people |
| Tiredness (lack of energy). Please choose the number that best describes how you feel right now, where 0 is “Not tired” and 10 is “Worst possible tiredness” | Fatigue |
| Anxiety (feeling nervous). Please choose the number that best describes how you feel right now, where 0 is “Not anxious” and 10 is “Worst possible anxiety” | Anxiety |
| Depression (feeling sad). Please choose the number that best describes how you feel right now, where 0 is “Not depressed” and 10 is “Worst possible depression” | Depression |
| I consider myself to be a creative person. | Creative person |
| I am engaged in creative type work on a regular basis. | Creative work |
