# Supplementary material for: Pain as bad as you can imagine or extremely severe pain? A randomized controlled trial comparing two pain scale anchors
Source: J Patient Rep Outcomes. 2023 Nov 29;7:123. doi: 10.1186/s41687-023-00665-w (PMC10686922; doi:10.1186/s41687-023-00665-w)
Supplement: Supplementary file 2 — Supplementary Material 2: Supplementary Table 2. Body Part where participants have pain. Data presented as frequency (percentage) [file 41687_2023_665_MOESM2_ESM.docx]

**Supplementary Table 2.** Body Part where participants have pain. Data presented as frequency (percentage).

|  |  | **Extreme,**  N = 405 | **Imagine,**  N = 424 |
| --- | --- | --- | --- |
| Head and/or face (Left) |  | 119 (29%) | 134 (32%) |
| Head and/or face (Right) |  | 114 (28%) | 134 (32%) |
| Neck (Left) |  | 208 (51%) | 205 (48%) |
| Neck (Right) |  | 205 (51%) | 205 (48%) |
| Shoulder (Left) |  | 170 (42%) | 170 (40%) |
| Shoulder (Right) |  | 188 (46%) | 188 (44%) |
| Upper arm (Left) |  | 70 (17%) | 72 (17%) |
| Upper arm (Right) |  | 80 (20%) | 77 (18%) |
| Elbow (Left) |  | 51 (13%) | 57 (13%) |
| Elbow (Right) |  | 66 (16%) | 64 (15%) |
| Underarm (Left) |  | 27 (6.7%) | 23 (5.4%) |
| Underarm (Right) |  | 23 (5.7%) | 32 (7.5%) |
| Hand (Left) |  | 143 (35%) | 157 (37%) |
| Hand (Right) |  | 158 (39%) | 159 (38%) |
| Chest (Left) |  | 52 (13%) | 58 (14%) |
| Chest (Right) |  | 41 (10%) | 56 (13%) |
| Side of the chest (Left) |  | 43 (11%) | 50 (12%) |
| Side of the chest (Right) |  | 43 (11%) | 48 (11%) |
| Abdomen (Left) |  | 70 (17%) | 84 (20%) |
| Abdomen (Right) |  | 73 (18%) | 82 (19%) |
| Sexual organs and/or groin area (Left) |  | 51 (13%) | 52 (12%) |
| Sexual organs and/or groin area (Right) |  | 49 (12%) | 48 (11%) |
| Upper back (Left) |  | 153 (38%) | 160 (38%) |
| Upper back (Right) |  | 157 (39%) | 161 (38%) |
| Lower back (Left) |  | 291 (72%) | 278 (66%) |
| Lower back (Right) |  | 286 (71%) | 281 (66%) |
| Hip and/or buttocks (Left) |  | 195 (48%) | 183 (43%) |
| Hip and/or buttocks (Right) |  | 196 (48%) | 208 (49%) |
| Thigh (Left) |  | 78 (19%) | 81 (19%) |
| Thigh (Right) |  | 74 (18%) | 85 (20%) |
| Knee (Left) |  | 168 (41%) | 168 (40%) |
| Knee (Right) |  | 163 (40%) | 165 (39%) |
| Lower leg (Left) |  | 97 (24%) | 108 (25%) |
| Lower leg (Right) |  | 103 (25%) | 105 (25%) |
| Foot (Left) |  | 160 (40%) | 170 (40%) |
| Foot (Right) |  | 159 (39%) | 168 (40%) |
| No Pain Location Selected |  | 0 (0%) | 1 (0.2%) |
