# Supplementary material for: Pain as bad as you can imagine or extremely severe pain? A randomized controlled trial comparing two pain scale anchors
Source: J Patient Rep Outcomes. 2023 Nov 29;7:123. doi: 10.1186/s41687-023-00665-w (PMC10686922; doi:10.1186/s41687-023-00665-w)
Supplement: Supplementary file 3 — Supplementary Material 3: Supplementary Table 3a. Dichotomized responses to pain questions based on anchor group. (“Extreme” corresponds to the changed anchor text “Extremely severe pain”; “Imagine” corresponds to the original anchor text “Pain as bad as you can imagine”). Data presented as frequency (percentage). Supplementary Table 3b. Categorized responses to pain questions based on anchor group. (“Extreme” corresponds to the changed anchor text “Extremely severe pain”; “Imagine” corresponds to the original anchor text “Pain as bad as you can imagine”). Data presented as frequency (percentage). Supplementary Table 3c. Responses to pain questions based on anchor group (“Extreme” corresponds to the changed anchor text “Extremely severe pain”; “Imagine” corresponds to the original anchor text “Pain as bad as you can imagine”). Data presented median (quartiles) [file 41687_2023_665_MOESM3_ESM.docx]

**Supplementary Table 3a**. Dichotomized responses to pain questions based on anchor group
(“*Extreme*” corresponds to the changed anchor text “*Extremely severe pain*”; “*Imagine*” corresponds to the original anchor text “*Pain as bad as you can imagine*”). Data presented as frequency (percentage).

|  | **Extreme,**  **N = 405** | **Imagine,**  **N = 424** | **p-value** |
| --- | --- | --- | --- |
| Worst ≥ 9 | 46 (11%) | 43 (10%)^1^ | 0.6 |
| Least ≤ 7 | 387 (96%) | 410 (97%)^1^ | 0.3 |
| Average ≥ 8 | 36 (8.9%)^1^ | 30 (7.1%)^1^ | 0.3 |
| Right now ≥ 8 | 47 (12%) | 52 (12%)^2^ | 0.8 |
| Troubling = 10 | 62 (15%) | 62 (15%) | 0.8 |

^1^1 unknown response. ^2^2 unknown responses

**Supplementary Table 3b.** Categorized responses to pain questions based on anchor group

(“*Extreme*” corresponds to the changed anchor text “*Extremely severe pain*”; “*Imagine*” corresponds to the original anchor text “*Pain as bad as you can imagine*”). Data presented as frequency (percentage).

|  | **Extreme,**  **N = 405** | **Imagine,**  **N = 424** | **p-value** |
| --- | --- | --- | --- |
| Worst |  |  | 0.7 |
| [0, 1] | 8 (2.0%) | 5 (1.2%) |  |
| [2, 4] | 60 (15%) | 76 (18%) |  |
| 5 | 46 (11%) | 49 (12%) |  |
| [6,8] | 245 (60%) | 250 (59%) |  |
| [9, 10] | 46 (11%) | 43 (10%) |  |
| Unknown | 0 | 1 |  |
| Least |  |  | 0.4 |
| [0, 1] | 101 (25%) | 129 (30%) |  |
| [2, 4] | 215 (53%) | 216 (51%) |  |
| 5 | 31 (7.7%) | 28 (6.6%) |  |
| [6,8] | 52 (13%) | 46 (11%) |  |
| [9, 10] | 6 (1.5%) | 4 (0.9%) |  |
| Unknown | 0 | 1 |  |
| Average |  |  | 0.5 |
| [0, 1] | 26 (6.4%) | 19 (4.5%) |  |
| [2, 4] | 155 (38%) | 181 (43%) |  |
| 5 | 68 (17%) | 76 (18%) |  |
| [6,8] | 147 (36%) | 137 (32%) |  |
| [9, 10] | 8 (2.0%) | 10 (2.4%) |  |
| Unknown | 1 | 1 |  |
| Right now |  |  | 0.3 |
| [0, 1] | 64 (16%) | 52 (12%) |  |
| [2, 4] | 153 (38%) | 173 (41%) |  |
| 5 | 42 (10%) | 57 (14%) |  |
| [6,8] | 131 (32%) | 123 (29%) |  |
| [9, 10] | 15 (3.7%) | 17 (4.0%) |  |
| Unknown | 0 | 2 |  |
| Troubling |  |  | 0.7 |
| [0, 1] | 1 (0.2%) | 2 (0.5%) |  |
| [2, 4] | 33 (8.1%) | 47 (11%) |  |
| 5 | 25 (6.2%) | 25 (5.9%) |  |
| [6,8] | 210 (52%) | 209 (49%) |  |
| [9, 10] | 136 (34%) | 141 (33%) |  |

**Supplementary Table 3c.** Responses to pain questions based on anchor group (“*Extreme*” corresponds to the changed anchor text “*Extremely severe pain*”; “*Imagine*” corresponds to the original anchor text “*Pain as bad as you can imagine*”). Data presented median (quartiles).

|  | **Extreme,**  **N = 405** | **Imagine,**  **N = 424** | **p-value** |
| --- | --- | --- | --- |
| Worst | 7 (5, 8) | 7 (5, 8)^1^ | 0.2 |
| Least | 3 (2, 4) | 3 (1, 4)^1^ | 0.14 |
| Average | 5 (3, 6)^1^ | 5 (3, 6)^1^ | 0.5 |
| Right now | 4 (2, 6) | 4 (2, 6)^2^ | >0.9 |
| Troubling | 8 (6, 9) | 8 (6, 9) | 0.7 |

^1^1 unknown response. ^2^2 unknown responses
