# Supplementary material for: Pain as bad as you can imagine or extremely severe pain? A randomized controlled trial comparing two pain scale anchors
Source: J Patient Rep Outcomes. 2023 Nov 29;7:123. doi: 10.1186/s41687-023-00665-w (PMC10686922; doi:10.1186/s41687-023-00665-w)
Supplement: Supplementary file 4 — Supplementary Material 4: Supplementary Table 4. Correlation between pain questions and age and sex questions based on anchor group (“Extreme” corresponds to the changed anchor text “Extremely severe pain”; “Imagine” corresponds to the original anchor text “Pain as bad as you can imagine”) [file 41687_2023_665_MOESM4_ESM.docx]

**Supplementary Table 4.** Correlation between pain questions and age and sex questions based on anchor group (“*Extreme*” corresponds to the changed anchor text “*Extremely severe pain*”; “*Imagine*” corresponds to the original anchor text “*Pain as bad as you can imagine*”).

|  | **Age** | | **Sex** | |
| --- | --- | --- | --- | --- |
|  | **Extreme** | **Imagine** | **Extreme** | **Imagine** |
| Worst | -0.06 | 0.04 | 0.09 | 0.06 |
| Least | 0.00 | 0.02 | 0.04 | -0.03 |
| Average | -0.03 | 0.04 | 0.04 | 0.05 |
| Right now | -0.10 | -0.04 | 0.07 | 0.04 |
