# Supplementary material for: Pain as bad as you can imagine or extremely severe pain? A randomized controlled trial comparing two pain scale anchors
Source: J Patient Rep Outcomes. 2023 Nov 29;7:123. doi: 10.1186/s41687-023-00665-w (PMC10686922; doi:10.1186/s41687-023-00665-w)
Supplement: Supplementary file 5 — Supplementary Material 5: Supplementary Table 5. Interaction term from linear regression model with pain as the outcome, primary predictors of creativity score, anchor group (“Extreme” corresponds to the changed anchor text “Extremely severe pain”; “Imagine” corresponds to the original anchor text “Pain as bad as you can imagine”), and interaction between anchor group and creativity score [file 41687_2023_665_MOESM5_ESM.docx]

**Supplementary Table 5.** Interaction term from linear regression model with pain as the outcome, primary predictors of creativity score, anchor group (“*Extreme*” corresponds to the changed anchor text “*Extremely severe pain*”; “*Imagine*” corresponds to the original anchor text “*Pain as bad as you can imagine*”), and interaction between anchor group and creativity score.

| **Pain** | **Predictor** | **β** | **95% CI** | **p-value** |
| --- | --- | --- | --- | --- |
| Worst | Interaction (Anchor x Creativity) | -0.07 | -0.20, 0.06 | 0.3 |
| Least | Interaction (Anchor x Creativity) | -0.06 | -0.20, 0.07 | 0.4 |
| Average | Interaction (Anchor x Creativity) | -0.08 | -0.21, 0.05 | 0.2 |
| Right now | Interaction (Anchor x Creativity) | -0.08 | -0.24, 0.08 | 0.3 |
| Troubling | Interaction (Anchor x Creativity) | -0.03 | -0.15, 0.10 | 0.7 |
